# Deep Brain Stimulation for Movement Disorders in Spain: Temporal Trends, Complications, and Sex-Related Disparities (2002–2019)

**DOI:** 10.3390/healthcare14050672

**Published:** 2026-03-06

**Authors:** Víctor Gómez-Mayordomo, Jose J. Zamorano-León, David Carabantes-Alarcon, Valentín Hernández-Barrera, Ana Lopez-de-Andrés, Natividad Cuadrado-Corrales, Fernando Alonso-Frech, Ana Jiménez-Sierra, Rodrigo Jiménez-García

**Affiliations:** 1 Clinical Neurosciences Institute, Hospital Blua Sanitas Valdebebas, Fundación Sanitas Hospitales, 28042 Madrid, Spain; vagomez.pex@sanitas.es; 2 Department of Public Health and Maternal & Child Health, Faculty of Medicine, Universidad Complutense de Madrid, Instituto de Investigación Sanitaria del Hospital Clínico San Carlos (IdISSC), 28040 Madrid, Spain; dcaraban@ucm.es (D.C.-A.); mariancu@ucm.es (N.C.-C.); rodrijim@ucm.es (R.J.-G.); 3 Preventive Medicine and Public Health Teaching and Research Unit, Faculty of Health Sciences, Universidad Rey Juan Carlos, 28922 Madrid, Spain; valentin.hernandez@urjc.es; 4 Department of Public Health and Maternal & Child Health, Faculty of Pharmacy, Universidad Complutense de Madrid, Instituto de Investigación Sanitaria del Hospital Clínico San Carlos (IdISSC), 28040 Madrid, Spain; anailo04@ucm.es; 5 HM CINAC (Centro Integral de Neurociencias Abarca Campal), Hospital Universitario HM Puerta del Sur, Instituto de Investigación Sanitaria HM Hospitales, 28938 Madrid, Spain; faalonso.hmcinac@hmhospitales.com; 6 Faculty of Medicine, Universidad CEU San Pablo, 28003 Madrid, Spain; a.jimenez100@usp.ceu.es

**Keywords:** deep brain stimulation, Parkinson’s disease, postoperative complications, sex, hospitalization, comorbidity

## Abstract

**Background/Objectives:** This study aimed to describe temporal trends in deep brain stimulation (DBS) use for Parkinson’s disease (PD), essential tremor (ET), and dystonia; characterize patient age and sex distribution and comorbidity; assess postoperative complications and in-hospital mortality (IHM) after implantation and explantation; and explore sex-specific differences in utilization and outcomes. **Methods:** We conducted a retrospective nationwide population-based study using the Spanish National Hospital Discharge Database (RAE-CMBD) from 2002 to 2019. All hospital admissions with DBS implantation or explantation/revision and a diagnosis of PD, ET, or dystonia were identified. Sociodemographic variables, the Charlson Comorbidity Index (CCI), length of hospital stay (LOHS), postoperative complications, and IHM were analyzed across three calendar periods and stratified by diagnosis and sex. **Results:** A total of 4883 admissions for DBS electrode implantations and 497 admissions for DBS explantation/revision were recorded. PD accounted for 82.6% of implantations, followed by ET (11.2%) and dystonia (6.3%). DBS activity increased significantly over time, while median LOHS declined from 12 to 6 days for implantations and from 13 to 5 days for explantations. Overall IHM after implantation was 0.27%, decreasing to 0.05% in 2014–2019; IHM after explantation was 0.6%. Most hospitalizations had low comorbidity (CCI = 0 in 87.8%), although comorbidity increased over time. Men represented approximately 60% of procedures in PD and ET. Women with PD underwent DBS at older ages, despite similar LOHS and IHM. Postoperative complications were recorded in 14.6% of implantations, mainly hardware-related issues (5–6%) and infections (1–2%), whereas infections (33%) and mechanical problems (27%) predominated among explantations. **Conclusions:** DBS use in Spain has expanded substantially, with shorter hospital stays and very low in-hospital mortality. Sex-related differences in utilization are increasing, and hardware complications and infections remain the most frequent conditions associated with explantation. As complications were identified only during the same hospitalization as the DBS procedure, late post-discharge events are not captured and could be underestimated; patient-level risks cannot be derived.

## 1. Introduction

Movement disorders such as Parkinson’s disease (PD), essential tremor (ET), and dystonia are common chronic neurological conditions and a major cause of disability worldwide [1,2,3,4]. PD is the second most common neurodegenerative disease, and its global prevalence is projected to rise substantially in the coming decades as the age of the population increases [1,2]. ET is considered the most prevalent isolated tremor disorder, often leading to marked functional impairment [3]. Focal and generalized dystonias, although less common, can cause severe, long-lasting motor disability, pain, and social stigma [4]. Together, these disorders impose a considerable burden on patients, caregivers, and health systems in terms of loss of independence, reduced quality of life, and increased healthcare utilization and costs [5].

Deep brain stimulation (DBS) has become an established surgical treatment for selected patients with movement disorders whose symptoms are insufficiently controlled with pharmacological therapy. Since its introduction in the 1990s, DBS of the subthalamic nucleus or internal globus pallidus has been shown in randomized controlled trials and long-term observational studies to improve motor symptoms, reduce motor complications, and enhance quality of life in advanced PD compared with best medical therapy alone [6,7]. Thalamic (ventral intermediate nucleus) DBS is an effective option for medication-refractory ET, and pallidal DBS is an accepted treatment for certain forms of dystonia, including primary generalized and segmental dystonia and some monogenic dystonias [8,9,10]. International guidelines now recommend DBS as a key component of the therapeutic armamentarium for appropriately selected patients with PD, ET, and dystonia [11,12,13].

Despite its benefits, DBS is a complex and resource-intensive intervention that carries specific risks. Most common complications are largely related to the implanted hardware, such as lead malposition, migration or fracture, pulse-generator malfunction, and device or wound infection, whereas perioperative complications include intracranial hemorrhage, seizures, and systemic medical events [14,15]. These complications may necessitate surgical revision or explantation of the system and are associated with prolonged hospital stays, additional costs, and in rare cases, serious morbidity or mortality [16]. Reported complication rates vary widely across centres and studies, partly due to differences in patient selection, surgical technique, follow-up duration, and definitions of adverse events [15,17]. Most available data come from single-centre series or clinical trials, which typically involve relatively small, highly selected cohorts and may not fully reflect routine clinical practice.

Over the past three decades, the indications and use of DBS have progressively expanded, and patient selection criteria have evolved. Improvements in imaging, surgical targeting, and perioperative management, together with growing surgical experience, have likely facilitated safer procedures and may have encouraged the treatment of older patients and those with greater comorbidity in routine clinical practice [18,19]. At the same time, concerns have been raised about potential inequities in access to advanced therapies [20,21]. However, evidence on sex-related patterns in DBS use remains limited and heterogeneous, and robust population-based data stratified by underlying movement disorders are scarce.

Despite nearly three decades of DBS activity in Spain, there is a lack of nationwide data specifically addressing the hospital burden of DBS implantation and explantation for movement disorders and the associated postoperative complications. A comprehensive, diagnosis- and sex-specific description of patients undergoing DBS, their comorbidity burden, and the spectrum and temporal evolution of perioperative complications is essential to inform patient counselling, refine selection criteria, and anticipate resource needs. This is also relevant from a public health perspective, as the number of potential candidates for DBS is expected to increase in parallel with the projected rise in the prevalence of PD and other movement disorders [22,23,24].

According to this, administrative health databases and national hospital discharge registries provide an opportunity to overcome some of these limitations by enabling the analysis of large, unselected cohorts over long time periods. In Spain, the Spanish National Hospital Discharge Database (RAE-CMBD) compiles standardized information on virtually all discharges from public hospitals, including demographic variables (sex and age), diagnoses, procedures, length of hospital stay, and in-hospital mortality [25,26].

In this context, we conducted a retrospective, nationwide, population-based study using the RAE-CMBD to characterize hospital admissions for DBS implantations and explantations or surgical revision due to movement disorders in Spain from 2002 to 2019. Our objectives were to: (1) describe long-term temporal trends in the use of DBS for PD, ET, and dystonia; (2) define the demographic and clinical profile of hospitalizations undergoing DBS, including age and comorbidity patterns; (3) quantify the frequency and evolution over time of postoperative complications and in-hospital mortality after DBS implantation and explantation; and (4) explore sex-specific differences in DBS utilization and outcomes, particularly among hospitalizations with PD and ET.

## 2. Materials and Methods

### 2.1. Study Design and Data Source

We conducted a retrospective, nationwide, population-based observational study using data from the Spanish National Hospital Discharge Database (*Registro de Altas del Conjunto Minimo Basico de Datos*; RAE-CMBD). This database is managed by the Spanish Ministry of Health and compiles information on virtually all discharges from public hospitals in Spain, covering more than 95% of all hospital admissions in the national health system. The RAE-CMBD includes demographic variables such as sex and date of birth, admission and discharge dates, discharge destination (e.g., home, death, transfer), and multiple diagnosis (up to 20) and procedure (up to 20) fields coded according to the Spanish adaptations of the International Classification of Diseases (ICD). A detailed description of the design, coverage, and data collection procedures of this registry has been published by the Ministry of Health [25].

During the study period, diagnoses and procedures were coded using ICD-9-CM until 2015 and ICD-10-ES from 2016 to 2019, following the transition previously described [25,26].

### 2.2. Study Population

We identified all hospitalizations recorded in the RAE-CMBD between 1 January 2002 and 31 December 2019 in which a DBS procedure for a movement disorder was coded. Two cohorts of admissions were defined: hospitalizations with codes indicating DBS electrode implantation and hospitalizations with codes indicating explantation, removal, or revision of existing DBS hardware.

To restrict the analysis to DBS procedures performed for movement disorders, we included only those admissions in which at least one diagnostic field contained a code compatible with Parkinson’s disease, essential tremor, or dystonia, using ICD-9-CM and ICD-10-ES codes that have been previously applied in administrative data analyses of Parkinsonian disorders [26]. No age restriction was applied.

Because the RAE-CMBD is an anonymized discharge-level database and does not include unique patient identifiers, multiple hospitalizations from the same individual cannot be linked. Consequently, each eligible admission was analyzed as a separate event and reported per hospitalization episode, reflecting the hospital burden associated with DBS implantation and explantation/revision procedures, as done in previous national studies based on this registry. We restricted the study period to 2002–2019 to avoid COVID-19-related disruptions in hospital activity from 2020 onwards, which could artefactually affect temporal trends.

### 2.3. Variables

For each hospitalization, we obtained sociodemographic information (age at admission and sex), clinical data (type of movement disorder and comorbidity burden), and outcomes related to the hospital stay.

Age was treated both as a continuous variable and categorized into five groups (<40, 40–49, 50–59, 60–69, and ≥70 years), following the stratification used in our tables to describe the age distribution of patients undergoing DBS implantation and explantation/revision. Sex was classified as male or female according to the RAE-CMBD record.

The diagnosis of the type of movement disorder was classified into three mutually exclusive categories: Parkinson’s disease, essential tremor, or dystonia. This classification was based on the presence of the corresponding ICD codes in any diagnostic position. In those few admissions in which more than one of these diagnoses co-occurred, a hierarchical rule was applied to assign a single underlying condition for descriptive purposes, prioritizing Parkinson’s disease, then essential tremor, and finally dystonia.

Overall comorbidity was summarized using the Charlson Comorbidity Index (CCI), calculated from all diagnosis fields recorded at discharge with validated algorithms for ICD-9-CM and ICD-10 administrative data [27]. We categorized the CCI into three groups (0, 1, and ≥2) to describe the distribution of comorbidity burden among hospitalizations and its evolution over time. None of the movement disorder diagnoses are included in the CCI, and therefore, they were not considered in the calculation of the index.

For each admission, we calculated the length of hospital stay (LOHS) as the difference in days between admission and discharge dates. In-hospital mortality (IHM) was defined as any death occurring during the index hospitalization as recorded in the discharge status field of the RAE-CMBD.

### 2.4. Postoperative Complications

Postoperative complications were identified using specific diagnosis and procedure codes recorded during the same hospitalization in which the DBS procedure took place. For DBS implantation admissions, we examined hardware-related complications (such as electrode malposition or lead migration, mechanical failure or malfunction of the device, and a need for surgical revision of the system), infectious complications (including device or pocket infection, postoperative wound infection, central nervous system infection, and sepsis), neurological complications (intracranial hemorrhage, epileptic seizures, and delirium), and systemic complications (pneumonia, deep vein thrombosis, pulmonary embolism, and acute renal failure). We also assessed the need for blood transfusion and for mechanical ventilation as indicators of procedure-related complexity and resource use. These procedures and diagnoses were identified in any position of the database.

The same set of complications was evaluated for admissions involving DBS explantation or surgical revision. In this cohort, particular attention was paid to the presence of device infection and hardware malfunction, as these are common indications for DBS system removal reported in the literature.

The ICD-9-CM and ICD-10-ES codes used to define all diagnoses, procedures, and complications are listed in Supplementary Table S1.

### 2.5. Time Periods and Stratified Analyses

To examine long-term temporal trends while minimizing the impact of year-to-year variability, the 18-year study period was a priori divided into three consecutive 6-year intervals (2002–2007, 2008–2013, and 2014–2019). This grouping guaranteed sufficient numbers of procedures in each stratum, especially for less prevalent diagnoses and interventions, and allowed robust comparisons over time, including subgroup analyses by diagnosis and sex. All analyses were conducted separately for DBS implantation and DBS explantation/revision admissions. Within these two cohorts, results were stratified by underlying movement disorder and, for Parkinson’s disease and essential tremor, by sex, following the approach used in previous RAE-CMBD-based studies on Parkinson’s disease hospitalizations in Spain [26].

### 2.6. Statistical Analysis

We first performed a descriptive analysis of all hospitalizations for DBS implantation and explantation. Continuous variables are shown as mean with standard deviation (SD) or as median with interquartile range (IQR) according to their normal distribution (Kolmogorov–Smirnov test). Categorical variables are reported as absolute frequencies and percentages.

To compare the distribution of study variables, we used the Chi-square test, or the Fisher exact test when required, for categorical variables. For continuous variables with normal distribution, we applied the Student *t*-test or analysis of variance (ANOVA), whereas for non-normally distributed variables, we used the U of Mann–Whitney or Kruskal–Wallis tests. The methods used to assess potential changes in the distribution of the variables over time included the Cochran–Mantel–Haenszel statistic or Cochran–Armitage test for categorical variables and the linear regression *t*-test or Jonckheere–Terpstra test for continuous variables.

The RAE-CMBD is an anonymized administrative database that is curated and quality-controlled by the Spanish Ministry of Health prior to release. For this study, the Ministry provided an anonymized, cleaned record-level extract for the study period. The final analytic outputs (tables/figures and key counts) were reviewed by the investigator group to ensure internal consistency and accuracy.

All statistical tests were two-sided, and a *p*-value < 0.05 was considered statistically significant. Statistical analyses were conducted using SPSS version 29.0.2.0 (IBM Corp., Armonk, NY, USA).

### 2.7. Ethical Considerations

The RAE-CMBD is an anonymized administrative database that does not contain direct personal identifiers. According to Spanish legislation on biomedical research and data protection, the use of fully anonymized secondary data provided by public institutions does not require informed consent from patients and exemption of evaluation and approval by a research ethics committee is granted [28]. Access to the RAE-CMBD can be obtained from the Spanish Ministry of Health upon submission of a formal data request [29].

## 3. Results

### 3.1. Characteristics and Temporal Trends of DBS Implantations

From 2002 to 2019, a total of 4883 hospital admissions for DBS electrode implantation due to movement disorders were recorded in Spain. The number of implantations increased steadily across the three time periods, from 1213 admissions in 2002–2007 to 1664 in 2008–2013 and 2006 in 2014–2019 (*p* < 0.001, Table 1).

PD was the main indication for DBS throughout the study, accounting for 4031 admissions (82.6%), followed by ET (545; 11.2%) and dystonia (307; 6.3%). The proportion of PD showed a modest decline over time (86.9% in 2002–2007 vs. 81.5% in 2014–2019; *p* < 0.001), whereas ET and dystonia increased slightly (ET 8.7% to 11.6%; dystonia 4.5% to 6.9%).

The mean age of hospitalizations undergoing DBS implantation for any movement disorder was 59.45 years (SD 11.67) and decreased slightly but significantly over the study period (60.27 years in 2002–2007 vs. 59.05 in 2014–2019; *p* = 0.009). Most procedures were performed in hospitalizations aged 60–69 years (43.2%), followed by those aged 50–59 years (24.9%). The proportion of hospitalizations aged ≥70 years declined from 18.2% in the first period to 13.6% in the last (*p* < 0.001).

Comorbidity burden, as measured by the grouped CCI, was generally low. Overall, 87.8% of admissions had a CCI of “0”, 9.7% had a CCI of “1”, and only 2.5% had a CCI “≥2”. However, there was a statistically significant increase in the proportion of hospitalizations with at least one comorbidity (CCI ≥ 1) over time (from 9.6% in 2002–2007 to 13.9% in 2014–2019; *p* = 0.012).

LOHS decreased markedly during the study period. The median LOHS for DBS implantation fell from 12 days (IQR 9) in 2002–2007 to 8 days (IQR 6) in 2008–2013 and 6 days (IQR 5) in 2014–2019 (*p* < 0.001). In parallel, IHM after DBS implantation declined significantly, from 0.82% in the first period to 0.12% in 2008–2013 and 0.05% in 2014–2019 (overall IHM 0.27%, *p* < 0.001 for trend).

### 3.2. DBS Implantation in Parkinson’s Disease According to Sex

**Figure 1:**
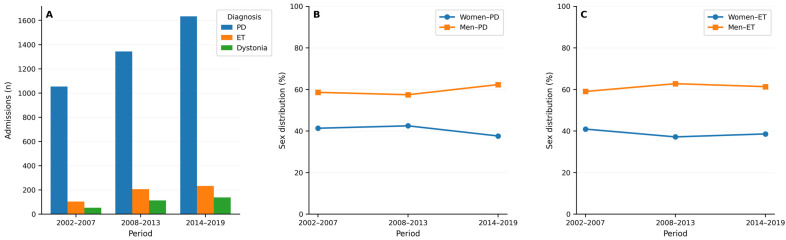
Temporal trends in DBS implantation volume by diagnosis (**A**) and sex among PD (**B**) and ET (**C**) cases across the three study periods. Footnote: PD, Parkinson’s disease. ET, essential tremor.

Among the 4031 DBS implantation admissions for PD, 2409 (59.8%) occurred in men and 1622 (40.2%) in women (Table 2 and Figure 1).

In men with PD, the number of implantations increased from 618 in 2002–2007 to 772 in 2008–2013 and 1019 in 2014–2019 (*p* < 0.001). Mean age remained stable around 60 years (60.8, 60.28, and 59.26 years in the three periods; *p* = 0.087), with most procedures performed in the 60–69-year group (44.1% overall). The proportion of men aged ≥70 years showed a modest decrease over time (16.2% to 10.5%; *p* = 0.020 for age categories). Comorbidity burden was low: 87.1% of male admissions had CCI = 0, 10.3% CCI = 1, and 2.6% CCI ≥ 2, with no statistically significant change in this distribution over time (*p* = 0.067). Median LOHS decreased from 12 days (IQR 10) to 8 days (IQR 6) and 6 days (IQR 5) (*p* < 0.001). IHM in men with PD was 0.25% overall (six deaths), with a clear decrease over time, although not significant (*p* = 0.070).

In women with PD, the number of implantations also increased steadily (from 436 to 571 and 615 admissions across the three periods; *p* < 0.001). Women were slightly older than men (overall mean age 61.9 vs. 60.0 years, *p* = 0.038), and half of the procedures were performed in those aged 60–69 years (51.3%). The proportion aged ≥70 years declined from 18.6% to 13.7% (*p* = 0.179 for age categories). Comorbidity was very low: 91.7% of female admissions had CCI = 0, and only 1.5% had CCI ≥ 2, without significant variation over time (*p* = 0.254). As in men, LOHS decreased significantly in women (median 12, 8, and 7 days; *p* < 0.001). IHM was 0.25% overall (four deaths), and a significant decline was observed across periods (*p* = 0.004).

### 3.3. DBS Implantation in Essential Tremor According to Sex

A total of 545 DBS implantation admissions were identified for ET, of which 335 (61.5%) occurred in men and 210 (38.5%) in women (Table 3 and Figure 1).

In men with ET, the number of implantations rose from 62 in 2002–2007 to 130 in 2008–2013 and 143 in 2014–2019 (*p* < 0.001). Mean age was around 62 years and remained stable (61.39, 62.37, and 62.54 years; *p* = 0.337). Across the whole period, 38.4% of male implantations were in the 60–69-year group and 27.6% in those ≥70 years. Comorbidity burden was modest, with 75.5% of admissions having CCI = 0 and 6.0% CCI ≥ 2; no significant changes in comorbidity distribution over time were observed (*p* = 0.522). Median LOHS decreased from 9.5 days (IQR 5) to 7 (IQR 7) and 5 (IQR 4) (*p* = 0.035). IHM in men with ET was very low (0.3%, 1 death) and did not change significantly over time (*p* = 0.110).

In women with ET, the total number of implantations increased from 43 to 77 and 90 across the three periods (*p* < 0.001). More than one-third of female implantations were performed in patients aged ≥70 years (36.7%) and 29.5% in those aged 60–69 years. Comorbidity remained low and stable over time (CCI = 0 in 78.1%, CCI ≥ 2 in 3.8%; *p* = 0.680). However, LOHS decreased markedly, from a median of 14 days (IQR 8) in 2002–2007 to 8 days (IQR 6) in 2008–2013 and 5 days (IQR 4) in 2014–2019 (*p* < 0.001). IHM was 0.95% (two deaths), with no significant temporal variation (*p* = 0.402).

Overall, these results indicate that DBS for ET was performed in slightly older hospitalizations than for PD, with a sizeable proportion of procedures in individuals aged ≥70 years, but with similarly low comorbidity and in-hospital mortality.

### 3.4. Postoperative Complications After DBS Implantation

During the study period, 713 of the 4883 DBS implantation admissions (14.6%) had at least one coded postoperative complication (Table 4). The distribution of underlying diagnoses among these complicated admissions was similar to that of the full cohort: 81.9% PD, 11.4% ET, and 6.7% dystonia, with no significant change over time.

Among all DBS implantations, the most frequent specific complications were electrode malposition or mechanical failure in 265 admissions (5.43%), device infection in 77 (1.58%), intracranial hemorrhage in 40 (0.82%), pneumonia in 20 (0.41%), and epileptic seizures in 52 (1.06%). The need for mechanical ventilation was recorded in 46 admissions (0.94%) and blood transfusion in 30 (0.61%).

**Figure 2:**
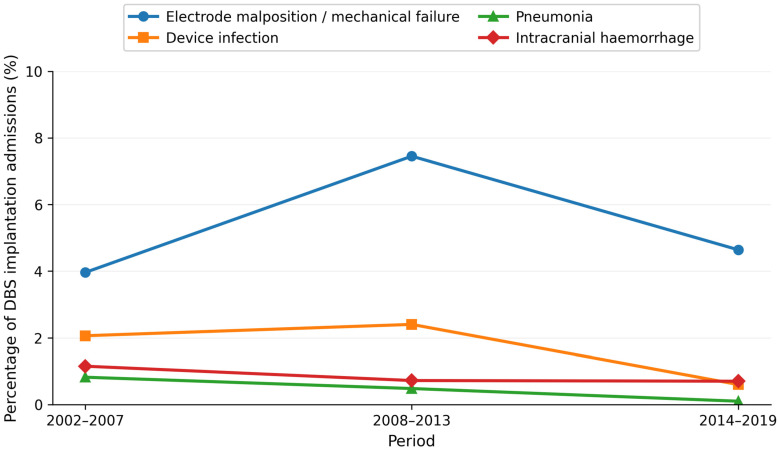
Temporal trends in most clinically relevant postoperative complications after DBS implantation.

Temporal patterns varied by complication. Rates of electrode malposition or mechanical failure peaked in 2008–2013 (7.45%) and were lower in the first and last periods (3.96% and 4.64%; *p* < 0.001). Device infection was more frequent in the first two periods (2.06% and 2.40%) and decreased to 0.60% in 2014–2019 (*p* < 0.001). Pneumonia also declined significantly over time (0.82%, 0.48%, and 0.10%; *p* = 0.007). The frequency of epileptic seizures showed a modest but statistically significant decrease (1.57%, 0.60%, and 1.15%; *p* = 0.040). For most other complications, absolute numbers were very low, and temporal differences did not reach statistical significance (Figure 2).

When complication rates were examined by underlying diagnosis (Supplementary Table S2), hardware-related problems and device infection were similarly frequent across PD, ET, and dystonia. For example, electrode malposition or mechanical failure affected 5.6% of PD admissions, 5.1% of ET, and 3.6% of dystonia admissions, with no significant differences between groups. However, pneumonia was significantly more common in ET than in PD (0.92% vs. 0.30%; *p* = 0.026), and epileptic seizures, blood transfusion, and mechanical ventilation occurred slightly more often in patients with dystonia, although absolute numbers were small. Also, several complications, such as ischemic stroke and blood transfusion, were rare (<1% of implantation admissions) and should be interpreted cautiously as admission-level coded events rather than definitive procedure-attributable complications.

### 3.5. Characteristics of DBS Explantations and Associated Complications

Over the same period, 497 hospital admissions for DBS surgical revisions or explantation/revision were identified (Table 5). The number of procedures increased from 82 in 2002–2007 to 131 in 2008–2013 and 284 in 2014–2019. Most explant admissions occurred in patients hospitalized with PD (425; 85.5%), followed by ET (38; 7.7%) and dystonia (34; 6.8%). Men represented 64.0% of explant admissions.

The mean age of hospitalizations undergoing DBS surgical revision was 62.3 years (SD 11.5) and did not change significantly over time (61.39, 62.37, and 62.54 years in the three periods; *p* = 0.790). Overall, 38.4% of admissions concerned patients aged 60–69 years and 27.6% of those aged ≥70 years. Comorbidity burden remained low but showed a significant temporal shift: the proportion of admissions with CCI = 0 decreased from 93.9% to 82.0%, whereas those with CCI = 1 increased from 2.4% to 14.1% (*p* = 0.007). Admissions with CCI ≥ 2 remained infrequent (3.0% overall).

As observed for implantations, LOHS for surgical revisions shortened over time. Median LOHS decreased from 13 days (IQR 10) in 2002–2007 to 7 days (IQR 11) in 2008–2013 and 5 days (IQR 8) in 2014–2019 (*p* < 0.001). IHM after DBS explantation/revision was 0.6% (three deaths, one in each period), with no significant differences across periods (*p* = 0.646).

Regarding complications specifically associated with explantation/revision (Supplementary Table S3), device infection was recorded in 165 admissions (33%) and electrode malposition or mechanical failure in 125 (27%), confirming these as the leading conditions associated with DBS system change or removal. Postoperative wound infection was present in 7.8% of explant admissions, while systemic complications such as pneumonia, deep vein thrombosis, pulmonary embolism, and acute renal failure were uncommon (each < 3%). Overall complication patterns were similar across PD, ET, and dystonia; although some comparisons (e.g., deep vein thrombosis and pulmonary embolism) reached statistical significance, the absolute number of events was very small, and no consistent diagnosis-specific gradient was observed.

## 4. Discussion

In this nationwide, population-based study using the Spanish RAE-CMBD, we describe temporal trends, clinical characteristics, and in-hospital outcomes of hospital admissions undergoing DBS for movement disorders in Spain from 2002 to 2019. We observed a steady increase in DBS implantations and explantations over time, with Parkinson’s disease as the predominant indication, followed by essential tremor and dystonia. Overall comorbidity and age increased modestly across the study period, whereas LOHS shortened and IHM decreased and remained very low. Hardware-related complications and infections were the main causes of explantation. Additionally, we found consistent sex differences, with men being more frequently hospitalized for DBS and women with PD undergoing surgery approximately two years later in life than men, despite similar comorbidity profiles and in-hospital outcomes.

These results are primarily representative of DBS-related hospital admissions within Spain and the Spanish healthcare system; extrapolation to other countries or healthcare contexts should be made with caution due to differences in referral pathways, reimbursement structures, surgical organization, and coding practices. However, our findings are consistent with the international literature reporting a sustained increase in DBS utilization for movement disorders over the last two decades. Large administrative database analyses from the United States, using the Nationwide Inpatient Sample and other national datasets, have shown a marked rise in the number of DBS procedures since the mid-1990s, accompanied by an expansion of indications and a gradual shift towards older and more comorbid patients. In these studies, PD was also the leading indication for DBS, followed by ET and dystonia, with PD typically representing 60–80% of procedures [30,31]. Data from Europe and Asia similarly report that PD is the most frequent indication, with ET and dystonia accounting for smaller but clinically relevant subsets [16,32,33,34,35]. Notably, studies from Latin America show that dystonia is the second most common indication after Parkinson’s disease, ahead of essential tremor [36]. Taken together, the diagnostic distribution and broad temporal trends we describe in Spain mirror those observed in other high-income countries.

Within this overall pattern, we observed a modest change in the relative contribution of diagnoses across periods. Interestingly, the relative share of PD among DBS implantations decreased modestly in the last study period, from 86.9% in 2002–2007 to 81.5% in 2014–2019, despite a continued increase in the absolute number of PD procedures. In parallel, the proportions of ET and dystonia rose slightly. A plausible interpretation is that, over the same period, access to other device-aided second-line therapies for advanced PD, such as continuous levodopa–carbidopa intestinal gel or subcutaneous apomorphine infusion, has progressively expanded in Spain [37]. These infusion therapies are often considered in older or more comorbid patients who might previously have been referred for DBS, and their wider availability could have shifted part of this population away from surgery, thereby reducing the proportion of PD DBS in the oldest and most comorbid groups. In the future, it will be important to examine how DBS utilization patterns evolve in the post-COVID era and in the context of the growing use of magnetic resonance-guided focused ultrasound (MRgFUS) for tremor and, more recently, for selected Parkinsonian symptoms, as these developments may further reshape the balance between DBS and other advanced therapies.

The clinical profile of hospital admissions undergoing DBS also resembles that reported elsewhere. The mean age at implantation in our cohort falls within the sixth to seventh decade of life, consistent with previous studies [16,30,38]. Over time, we observed a progressive increase in the CCI, indicating that a growing proportion of hospitalizations had at least one relevant comorbidity. The absolute number of hospitalizations of patients older than 70 years also increased, although the overall proportion lowered due to a greater rise in younger patients. Importantly, despite this higher comorbidity burden, we found that LOHS decreased over time and IHM remained very low for both implantation and explantation/revision procedures. This pattern is consistent with international data showing that, as surgical and perioperative techniques have evolved, DBS is increasingly being offered to older and more comorbid patients, with low perioperative mortality and acceptable morbidity even in carefully selected older individuals [38,39,40]. In Spain, previous nationwide analyses of overall PD hospitalizations have shown a parallel trend of increasing comorbidity, with stable or only slightly increased adjusted in-hospital mortality and a progressive reduction in LOHS [26]. Taken together, these data support patient decision-making, as current mortality related to DBS in Spain remains below 0.1%.

The sex differences observed in our cohort deserve special consideration. Men accounted for most DBS procedures and hospitalizations across all diagnostic groups, and this “gender gap” widened steadily over the study period. Women with PD underwent surgery two years later in life than men but did not show worse in-hospital outcomes or longer stays, suggesting that this delay is not explained by a higher perioperative risk as captured in our data. This pattern replicates the “gender gap” described in other countries. Jost et al. described sex differences in PD surgery, showing that women are less likely to receive DBS and often present with more advanced disability at the time of implantation, despite similar postoperative benefits [20]. The recent literature on disparities in DBS and other advanced therapies for PD has also highlighted intersectional barriers related to sex, socioeconomic status, and race, which may influence referral patterns, patient preferences, and decision-making [21]. Other factors may include gender differences in health-seeking behaviour, caregiver roles, risk perception, and potential bias in referral and selection processes [41,42]. Moreover, women with PD may experience a greater burden of non-motor symptoms and quality-of-life impairment, which can further modulate expectations and priorities regarding surgery [43,44,45].

Our findings may suggest disparities in access; however, several alternative explanations should be considered. First, male predominance in DBS may partly reflect the higher prevalence of PD in men (roughly two-fold in many epidemiological studies) and a less marked, but still present, male predominance in ET [2,3]. Second, differences in referral patterns and care pathways may play a role: for example, neurologists might be more likely to refer younger, working-age men when motor disability interferes with employment, while women might be referred later or less often [21,41,42]. Finally, unmeasured comorbidities not captured by the CCI—such as cognitive impairment, psychiatric symptoms, frailty, or limited social support—might differentially affect eligibility or timing of DBS in women and men. Future studies should integrate clinical, psychosocial, and qualitative data to better understand sex- and gender-related determinants of DBS access and timing in Spain, and to design interventions that promote equitable care.

Complications, particularly those related to hardware, are a critical dimension when evaluating DBS programs. In our study, around 14% of implantation admissions were complicated by at least one coded adverse event, and approximately 8% of surgeries were associated with device-related mechanical problems or infections. These proportions are broadly in line with large single-centre series and systematic reviews, which have reported overall complication rates ranging from roughly 5% to 30% over variable follow-up periods, depending on how adverse events are defined and ascertained [14,15,46,47,48,49]. Large US database analyses have shown that between 15% and 34% of DBS procedures in routine practice are revisions or removals, with infection and hardware malfunction among the main drivers [50].

Electrode-related complications are consistent with other international studies that report rates of 5–10% [15,16,48]. In our series, hardware malposition or malfunction accounted for 5–6% of procedures and infections for 1–2%. We observed a transient increase in ‘electrode malposition/mechanical failure’ during 2008–2013, followed by a decline in 2014–2019. This pattern may reflect the rapid expansion of DBS activity during the mid-period, with greater heterogeneity in case mix and a potential learning-curve/centre-volume effect. In addition, because our dataset is episode-based, early lead revisions or staged procedures may be captured as separate admissions, inflating per-admission frequencies. Finally, temporal changes in postoperative imaging practices or coding sensitivity could have increased the detection and documentation of hardware-related events. Notably, this peak was not accompanied by increases in severe systemic complications or in-hospital mortality, suggesting a technical/reporting phenomenon rather than a generalized worsening of perioperative safety.

The infection proportion observed appears to lie at the lower end of the published range, although underestimation is likely, as some infections are probably managed in outpatient settings or during readmissions not coded with DBS procedure codes [17,49,51,52,53,54,55]. Device infections in our cohort accounted for only a minority of index implantations but were disproportionately represented among explantations and surgical revisions, suggesting that many infections are delayed rather than acute and ultimately culminate in hardware removal. Consistent with this, hardware-related complications and infections were frequently associated with explantation/revision in our cohort.

From a health-system perspective, the prominence of hardware-related complications and infections has important implications. Observational studies and expert-based recommendations have emphasized that DBS infections, although relatively infrequent, are associated with substantial morbidity, prolonged courses of intravenous and oral antibiotics, longer hospital stays, repeated procedures, and high costs, particularly when complete system explantation is required [17,49,51,52,53,54,55]. Our findings, showing that infections and mechanical complications are associated with explantation, support the prioritization of infection-prevention protocols, concentration of DBS surgery in experienced centres, and structured follow-up pathways as key quality and safety indicators for DBS programs in Spain.

Our study has several strengths. First, it is based on a nationwide administrative database that captures more than 95% of public hospital discharges in Spain, providing a comprehensive, real-world overview of DBS practice over nearly two decades. Second, the RAE-CMBD enables the analysis of a large sample of cases that would be impossible to obtain in other study settings. Third, we distinguished between DBS implantations and explantations/revisions, and stratified analyses by primary diagnosis and sex, allowing a nuanced characterization of trends and outcomes in PD, ET, and dystonia. Fourth, our long observation period spans major changes in DBS technology and perioperative care, enabling the description of temporal improvements in LOHS and stability of IHM despite increasing age and comorbidity.

Nevertheless, several limitations must be acknowledged. As with all studies based on administrative data, the RAE-CMBD lacks detailed clinical information such as disease duration, motor subtype, levodopa responsiveness, DBS target and programming parameters, cognitive status, or specific surgical techniques (awake vs. asleep, frame-based vs. frameless). In addition, the RAE-CMBD does not record ethnicity/race, country of birth, or other migration-background variables; therefore, we could not assess potential disparities in DBS access or outcomes across ethnic minority or migrant populations. The database also lacks information on referral pathways and waiting-list times (e.g., time from clinical indication to surgery), precluding analyses of waiting time trends by disorder. All these factors can influence both the indication for DBS and the risk of complications but could not be evaluated. Misclassification and under-coding are possible, particularly for specific complications and for secondary diagnoses that may not be systematically recorded [56]. The database is episode-based, so we could not track individual patients over time or capture long-term outcomes beyond index admission, including delayed complications (such as later infections), stimulation efficacy, or quality-of-life changes, which may underestimate the overall complication burden. Moreover, in-hospital mortality reflects deaths occurring during the index admission only and therefore underestimates post-discharge mortality. However, perioperative estimates from published DBS cohorts suggest that subsequent mortality is very low (approximately 0–0.3% at 30 days and ~0.2% at 90 days) [57,58]. For the same reason, we were unable to determine how many unique individuals underwent DBS during the study period or to calculate true complication, reintervention, or explantation rates at the patient level. Additionally, private hospitals and outpatient procedures are incompletely represented, which might lead to an underestimation of the true burden of complications and revisions. Finally, changes in coding systems (transition from ICD-9-CM to ICD-10-ES) over the study period might have introduced artefacts in temporal trends, although we applied consistent coding algorithms to identify diagnoses and procedures [26].

Finally, changes in coding systems (transition from ICD-9-CM to ICD-10-ES) over the study period might have introduced artefacts in temporal trends, even though we applied consistent coding algorithms to identify diagnoses and procedures [26]. Importantly, the coding era is almost completely aligned with calendar time; therefore, modelling the coding era as a covariate would be highly collinear with period effects and may yield unstable or difficult-to-interpret estimates. In addition, for some administrative endpoints (particularly complications and explantation/revision causes), the ICD-9 to ICD-10 transition does not always allow a straightforward one-to-one mapping, which may lead to differential misclassification across eras. For these reasons, we did not apply model-based “corrections” for the coding era, and temporal trends across the transition should be interpreted with caution. Similarly, because the dataset lacks key clinical variables required for meaningful perioperative risk adjustment and does not allow patient-level linkage across admissions, we did not perform risk-adjusted analyses, and our findings should be considered hypothesis-generating.

## 5. Conclusions

In conclusion, this nationwide study shows that DBS for movement disorders in Spain has expanded over the last two decades, with increasing age and comorbidity of treated patients, stable very low in-hospital mortality, and shorter hospital stays. Hardware complications and infections remain the most frequent diagnoses associated with explantation, and women with PD tend to undergo DBS less frequently and at an older age than men, despite similar in-hospital outcomes. These findings underscore the importance of optimizing patient selection, perioperative care, and infection-prevention strategies, while addressing potential sex- and gender-related inequities in access to DBS.

## Figures and Tables

**Table 1:** Characteristics of hospital admissions for deep brain stimulation electrode implants in movement disorders in Spain (2002–2019).

		2002–2007	2008–2013	2014–2019	Total	*p*-Value *
Total, N		1213	1664	2006	4883	<0.001
Diagnosis, N (%)	Parkinson’s disease	1054 (86.89)	1343 (80.71)	1634 (81.46)	4031 (82.55)	<0.001
	Essential tremor	105 (8.66)	207 (12.44)	233 (11.62)	545 (11.16)	
	Dystonia	54 (4.45)	114 (6.85)	139 (6.93)	307 (6.29)	
Age Mean (SD)		60.27 (11.51)	59.34 (11.93)	59.05 (11.54)	59.45 (11.67)	0.009
Age categories, N (%)	<40 years	70 (5.77)	122 (7.33)	106 (5.28)	298 (6.1)	<0.001
	40–49 years	100 (8.24)	179 (10.76)	222 (11.07)	501 (10.26)	
	50–59 years	307 (25.31)	382 (22.96)	526 (26.22)	1215 (24.88)	
	60–69 years	515 (42.46)	714 (42.91)	880 (43.87)	2109 (43.19)	
	≥70 years	221 (18.22)	267 (16.05)	272 (13.56)	760 (15.56)	
CCI, N (%)	0	1096 (90.35)	1463 (87.92)	1727 (86.09)	4286 (87.77)	0.012
	1	92 (7.58)	159 (9.56)	223 (11.12)	474 (9.71)	
	2 or more	25 (2.06)	42 (2.52)	56 (2.79)	123 (2.52)	
LOHS, Median (IQR)		12 (9)	8 (6)	6 (5)	8 (7)	<0.001
IHM, N (%)		10 (0.82)	2 (0.12)	1 (0.05)	13 (0.27)	<0.001

CCI, Charlson Comorbidity Index. LOHS, length of hospital stay. IQR, interquartile range. IHM, in-hospital mortality. * *p* value to assess time trend from 2002 to 2019.

**Table 2:** Characteristics of hospital admissions for deep brain stimulation electrode implants in Parkinson’s disease in Spain (2002–2019), according to sex.

		2002–2007	2008–2013	2014–2019	Total	*p*-Value *
Male						
Total, N		618	772	1019	2409	<0.001
Age Mean (SD)		60.8 (9.1)	60.28 (9.28)	59.26 (9.16)	59.98 (9.2)	0.087
Age categories, N (%)	<40 years	12 (1.94)	21 (2.72)	22 (2.16)	55 (2.28)	0.020
	40–49 years	59 (9.55)	92 (11.92)	138 (13.54)	289 (12)	
	50–59 years	185 (29.94)	207 (26.81)	300 (29.44)	692 (28.73)	
	60–69 years	262 (42.39)	348 (45.08)	452 (44.36)	1062 (44.08)	
	≥70 years	100 (16.18)	104 (13.47)	107 (10.5)	311 (12.91)	
Grouped Charlson Index, N (%)	0	554 (89.64)	670 (86.79)	875 (85.87)	2099 (87.13)	0.067
	1	46 (7.44)	81 (10.49)	121 (11.87)	248 (10.29)	
	2 or more	18 (2.91)	21 (2.72)	23 (2.26)	62 (2.57)	
LOHS, Median (IQR)		12 (10)	8 (6)	6 (5)	8 (7)	<0.001
IHM, N (%)		4 (0.65)	1 (0.13)	1 (0.1)	6 (0.25)	0.070
**Female**						
Total, N		436	571	615	1622	<0.001
Age Mean (SD)		62.61 (8.81)	61.67 (8.83)	61.63 (8.07)	61.91 (8.55)	0.211
Age Categories, N (%)	<40 years	5 (1.15)	15 (2.63)	7 (1.14)	27 (1.66)	0.179
	40–49 years	25 (5.73)	41 (7.18)	37 (6.02)	103 (6.35)	
	50–59 years	104 (23.85)	136 (23.82)	165 (26.83)	405 (24.97)	
	60–69 years	221 (50.69)	289 (50.61)	322 (52.36)	832 (51.29)	
	≥70 years	81 (18.58)	90 (15.76)	84 (13.66)	255 (15.72)	
Grouped Charlson Index, N (%)	0	407 (93.35)	529 (92.64)	552 (89.76)	1488 (91.74)	0.254
	1	24 (5.5)	34 (5.95)	51 (8.29)	109 (6.72)	
	2 or more	5 (1.15)	8 (1.4)	12 (1.95)	25 (1.54)	
LOHS, Median (IQR)		12 (9)	8 (6)	7 (5)	8 (7)	<0.001
IHM, N (%)		4 (0.92)	0 (0)	0 (0)	4 (0.25)	0.004

LOHS, length of hospital stay. IQR, interquartile range. IHM, in-hospital mortality. * *p* value to assess time trend from 2002 to 2019.

**Table 3:** Characteristics of hospital admissions for deep brain stimulation electrode implants in essential tremor in Spain (2002–2019), according to gender.

		2002–2007	2008–2013	2014–2019	Total	*p*-Value *
Male						
Total, N		62 (100)	130 (100)	143 (100)	335 (100)	<0.001
Age Mean (SD)		60.68 (14.73)	60.98 (14.65)	62.87 (10.68)	61.73 (13.12)	0.337
Age categories, N (%)	<40 years	8 (12.9)	14 (10.77)	6 (4.2)	28 (8.36)	0.388
	40–49 years	3 (4.84)	11 (8.46)	12 (8.39)	26 (7.76)	
	50–59 years	12 (19.35)	21 (16.15)	27 (18.88)	60 (17.91)	
	60–69 years	21 (33.87)	41 (31.54)	57 (39.86)	119 (35.52)	
	≥70 years	18 (29.03)	43 (33.08)	41 (28.67)	102 (30.45)	
CCI, N (%)	0	50 (80.65)	96 (73.85)	107 (74.83)	253 (75.52)	0.522
	1	11 (17.74)	26 (20)	25 (17.48)	62 (18.51)	
	2 or more	1 (1.61)	8 (6.15)	11 (7.69)	20 (5.97)	
LOHS, Median (IQR)		9.5 (5)	7 (7)	5 (4)	7 (6)	0.035
IHM, N (%)		1 (1.61)	0 (0)	0 (0)	1 (0.3)	0.110
**Female**						
Total, N		43 (100)	77 (100)	90 (100)	210 (100)	<0.001
Age Mean (SD)		59.93 (16.73)	58.09 (16.36)	63.26 (11.74)	60.68 (14.76)	0.132
Age categories, N (%)	<40 years	8 (18.6)	12 (15.58)	5 (5.56)	25 (11.9)	0.089
	40–49 years	5 (11.63)	13 (16.88)	8 (8.89)	26 (12.38)	
	50–59 years	2 (4.65)	6 (7.79)	12 (13.33)	20 (9.52)	
	60–69 years	9 (20.93)	21 (27.27)	32 (35.56)	62 (29.52)	
	≥70 years	19 (44.19)	25 (32.47)	33 (36.67)	77 (36.67)	
CCI, N (%)	0	35 (81.4)	60 (77.92)	69 (76.67)	164 (78.1)	0.680
	1	8 (18.6)	13 (16.88)	17 (18.89)	38 (18.1)	
	2 or more	0 (0)	4 (5.19)	4 (4.44)	8 (3.81)	
LOHS, Median (IQR)		14 (8)	8 (6)	5 (4)	7 (7)	<0.001
IHM, N (%)		1 (2.33)	1 (1.3)	0 (0)	2 (0.95)	0.402

CCI, Charlson Comorbidity Index. LOHS, length of hospital stay. IQR, interquartile range. IHM, in-hospital mortality. * *p* value to assess time trend from 2002 to 2019.

**Table 4:** Postoperative complications for deep brain stimulation electrode implants in movement disorders in Spain (2002–2019).

		2002–2007	2008–2013	2014–2019	Total	*p*-Value *
Total, N (%)		197 (100)	275 (100)	241 (100)	713 (100)	<0.001
Total by diagnosis, N (%)	Parkinson’s disease	164 (83.25)	228 (82.91)	192 (79.67)	584 (81.91)	0.538
	Essential tremor	21 (10.66)	33 (12)	27 (11.20)	81 (11.36)	0.899
	Dystonia	12 (6.09)	14 (5.09)	22 (9.13)	48 (6.73)	0.173
Electrode malposition/ mechanical failure, N (%)		48 (3.96)	124 (7.45)	93 (4.64)	265 (5.43)	<0.001
Device infection, N (%)		25 (2.06)	40 (2.4)	12 (0.6)	77 (1.58)	<0.001
Intracranial hemorrhage, N (%)		14 (1.15)	12 (0.72)	14 (0.7)	40 (0.82)	0.327
CNS infection, N (%)		0 (0)	1 (0.06)	5 (0.25)	6 (0.12)	0.098
Postoperative wound infection, N (%)		10 (0.82)	12 (0.72)	5 (0.25)	27 (0.55)	0.054
Sepsis, N (%)		0 (0)	2 (0.12)	1 (0.05)	3 (0.06)	0.422
Pneumonia, N (%)		10 (0.82)	8 (0.48)	2 (0.1)	20 (0.41)	0.007
Deep vein thrombosis, N (%)		2 (0.16)	2 (0.12)	0 (0)	4 (0.08)	0.227
Pulmonary thromboembolism, N (%)		1 (0.08)	3 (0.18)	0 (0)	4 (0.08)	0.164
Acute renal failure, N (%)		3 (0.25)	2 (0.12)	1 (0.05)	6 (0.12)	0.301
Delirium, N (%)		12 (0.99)	10 (0.6)	13 (0.65)	35 (0.72)	0.425
Epileptic seizures, N (%)		19 (1.57)	10 (0.6)	23 (1.15)	52 (1.06)	0.040
Blood transfusion, N (%)		7 (0.58)	7 (0.42)	16 (0.8)	30 (0.61)	0.341
Mechanical ventilation, N (%)		15 (1.24)	15 (0.9)	16 (0.8)	46 (0.94)	0.448

CNS, central nervous system. * *p* value to assess time trend from 2002 to 2019.

**Table 5:** Characteristics of hospital admissions for deep brain stimulation explantations/revisions in movement disorders in Spain (2002–2019).

		2002–2007	2008–2013	2014–2019	Total	*p*-Value *
Total, N		82	131	284	497	
Diagnosis, N (%)	Parkinson’s disease	73 (89.02)	115 (87.79)	237 (83.45)	425 (85.51)	0.421
	Essential tremor	3 (3.66)	10 (7.63)	25 (8.8)	38 (7.65)	
	Dystonia	6 (7.32)	6 (4.58)	22 (7.75)	34 (6.84)	
Sex, N (%)	Male	52 (63.41)	87 (66.41)	179 (63.03)	318 (63.98)	0.795
	Female	30 (36.59)	44 (33.59)	105 (36.97)	179 (36.02)	
Age Mean (SD)		61.39 (10.56)	62.37 (11.88)	62.54 (11.57)	62.3 (11.48)	0.790
Age categories, N (%)	<40 years	3 (3.66)	6 (4.58)	11 (3.87)	20 (4.02)	0.066
	40–49 years	7 (8.54)	5 (3.82)	27 (9.51)	39 (7.85)	
	50–59 years	27 (32.93)	34 (25.95)	49 (17.25)	110 (22.13)	
	60–69 years	24 (29.27)	50 (38.17)	117 (41.2)	191 (38.43)	
	≥70 years	21 (25.61)	36 (27.48)	80 (28.17)	137 (27.57)	
CCI, N (%)	0	77 (93.9)	120 (91.6)	233 (82.04)	430 (86.52)	0.007
	1	2 (2.44)	10 (7.63)	40 (14.08)	52 (10.46)	
	2 or more	3 (3.66)	1 (0.76)	11 (3.87)	15 (3.02)	
LOHS, Median (IQR)		13 (10)	7 (11)	5 (8)	7 (11)	<0.001
IHM, N (%)		1 (1.22)	1 (0.76)	1 (0.35)	3 (0.6)	0.646

CCI, Charlson Comorbidity Index. LOHS, length of hospital stay. IQR, interquartile range. IHM, in-hospital mortality. * *p* value to assess time trend from 2001 to 2019.

## Data Availability

According to the contract signed with the Spanish Ministry of Health and Social Services, which provided access to the databases from the Spanish National Hospital Database, we cannot share the databases with any other investigator, and we have to destroy the databases once the investigation has concluded. Consequently, we cannot upload the databases to any public repository. However, any investigator can apply for access to the databases by filling out the questionnaire available at https://www.mscbs.gob.es/estadEstudios/estadisticas/estadisticas/estMinisterio/SolicitudCMBDdocs/2018_Formulario_Peticion_Datos_RAE_CMBD.pdf (accessed on 19 December 2025). All other relevant data are included in the paper.

## Supplementary Materials

The following supporting information can be downloaded at https://www.mdpi.com/article/10.3390/healthcare14050672/s1, Table S1: ICD9 and ICD10 codes for diagnosis, procedures and complications used in this investigation; Table S2: Stratified postoperative complications for deep brain stimulation electrode implants in movement disorders in Spain according to diagnosis (2002–2019); Table S3: Stratified postoperative complications for deep brain stimulation explantations in movement disorders in Spain (2002–2019).

## Abbreviations

The following abbreviations are used in this manuscript:

PD	Parkinson’s disease
ET	Essential tremor
DBS	Deep brain stimulation
RAE-CMBD	Spanish National Hospital Discharge Database
ICD	International Classification of Diseases
CCI	Charlson Comorbidity Index
LOHS	Length of hospital stay
IHM	In-hospital mortality

## References

[1] GBD 2016 Neurology Collaborators (2019). Global, regional, and national burden of neurological disorders, 1990-2016: A systematic analysis for the Global Burden of Disease Study 2016. Lancet Neurol..

[2] Ben-Shlomo Y., Darweesh S., Llibre-Guerra J., Marras C., San Luciano M., Tanner C. (2024). The epidemiology of Parkinson’s disease. Lancet.

[3] Louis E.D., Ferreira J.J. (2010). How common is the most common adult movement disorder? Update on the worldwide prevalence of essential tremor. Mov. Disord..

[4] Albanese A., Bhatia K., Bressman S.B., Delong M.R., Fahn S., Fung V.S., Hallett M., Jankovic J., Jinnah H.A., Klein C. (2013). Phenomenology and classification of dystonia: A consensus update. Mov. Disord..

[5] Yang W., Hamilton J.L., Kopil C., Beck J.C., Tanner C.M., Albin R.L., Ray Dorsey E., Dahodwala N., Cintina I., Hogan P. (2020). Current and projected future economic burden of Parkinson’s disease in the U.S. npj Park. Dis..

[6] Deuschl G., Schade-Brittinger C., Krack P., Volkmann J., Schäfer H., Bötzel K., Daniels C., Deutschländer A., Dillmann U., Eisner W. (2006). A randomized trial of deep-brain stimulation for Parkinson’s disease. N. Engl. J. Med..

[7] Follett K.A., Weaver F.M., Stern M., Hur K., Harris C.L., Luo P., Marks W.J., Rothlind J., Sagher O., Moy C. (2010). Pallidal versus subthalamic deep-brain stimulation for Parkinson’s disease. N. Engl. J. Med..

[8] Kumar R., Lozano A.M., Sime E., Lang A.E. (2003). Long-term follow-up of thalamic deep brain stimulation for essential and parkinsonian tremor. Neurology.

[9] Kupsch A., Benecke R., Müller J., Trottenberg T., Schneider G.H., Poewe W., Eisner W., Wolters A., Müller J.U., Deuschl G. (2006). Pallidal deep-brain stimulation in primary generalized or segmental dystonia. N. Engl. J. Med..

[10] Vidailhet M., Vercueil L., Houeto J.L., Krystkowiak P., Lagrange C., Yelnik J., Bardinet E., Benabid A.L., Navarro S., Dormont D. (2007). Bilateral, pallidal, deep-brain stimulation in primary generalised dystonia: A prospective 3 year follow-up study. Lancet Neurol..

[11] Fox S.H., Katzenschlager R., Lim S.Y., Barton B., de Bie R.M.A., Seppi K., Coelho M., Sampaio C., Movement Disorder Society Evidence-Based Medicine Committee (2018). International Parkinson and movement disorder society evidence-based medicine review: Update on treatments for the motor symptoms of Parkinson’s disease. Mov. Disord..

[12] Zesiewicz T.A., Elble R.J., Louis E.D., Gronseth G.S., Ondo W.G., Dewey R.B., Okun M.S., Sullivan K.L., Weiner W.J. (2011). Evidence-based guideline update: Treatment of essential tremor: Report of the Quality Standards subcommittee of the American Academy of Neurology. Neurology.

[13] Albanese A., Asmus F., Bhatia K.P., Elia A.E., Elibol B., Filippini G., Gasser T., Krauss J.K., Nardocci N., Newton A. (2011). EFNS guidelines on diagnosis and treatment of primary dystonias. Eur. J. Neurol..

[14] Engel K., Huckhagel T., Gulberti A., Pötter-Nerger M., Vettorazzi E., Hidding U., Choe C.U., Zittel S., Braaß H., Ludewig P. (2018). Towards unambiguous reporting of complications related to deep brain stimulation surgery: A retrospective single-center analysis and systematic review of the literature. PLoS ONE.

[15] Jitkritsadakul O., Bhidayasiri R., Kalia S.K., Hodaie M., Lozano A.M., Fasano A. (2017). Systematic review of hardware-related complications of Deep Brain Stimulation: Do new indications pose an increased risk?. Brain Stimul..

[16] Jung I.H., Chang K.W., Park S.H., Chang W.S., Jung H.H., Chang J.W. (2022). Complications After Deep Brain Stimulation: A 21-Year Experience in 426 Patients. Front. Aging Neurosci..

[17] Kantzanou M., Korfias S., Panourias I., Sakas D.E., Karalexi M.A. (2021). Deep Brain Stimulation-Related Surgical Site Infections: A Systematic Review and Meta-Analysis. Neuromodulation.

[18] Isaacs B.R., Keuken M.C., Alkemade A., Temel Y., Bazin P.L., Forstmann B.U. (2020). Methodological Considerations for Neuroimaging in Deep Brain Stimulation of the Subthalamic Nucleus in Parkinson’s Disease Patients. J. Clin. Med..

[19] Jiang C., Wang J., Chen T., Li X., Cui Z. (2022). Short- and Long-Term Efficacy and Safety of Deep-Brain Stimulation in Parkinson’s Disease Patients aged 75 Years and Older. Brain Sci..

[20] Jost S.T., Strobel L., Rizos A., Loehrer P.A., Ashkan K., Evans J., Rosenkranz F., Barbe M.T., Fink G.R., Franklin J. (2022). Gender gap in deep brain stimulation for Parkinson’s disease. npj Park. Dis..

[21] Memon A.A., Gelman K., Melott J., Billings R., Fullard M., Catiul C., Miocinovic S., Amara A.W. (2023). A systematic review of health disparities research in deep brain stimulation surgery for Parkinson’s disease. Front. Hum. Neurosci..

[22] Dorsey E.R., Sherer T., Okun M.S., Bloem B.R. (2018). The Emerging Evidence of the Parkinson Pandemic. J. Park. Dis..

[23] Bach J.P., Ziegler U., Deuschl G., Dodel R., Doblhammer-Reiter G. (2011). Projected numbers of people with movement disorders in the years 2030 and 2050. Mov. Disord..

[24] Santos García D., Blázquez-Estrada M., Calopa M., Escamilla-Sevilla F., Freire E., García Ruiz P.J., Grandas F., Kulisevsky J., López-Manzanares L., Martínez Castrillo J.C. (2021). Present and Future of Parkinson’s Disease in Spain: PARKINSON-2030 Delphi Project. Brain Sci..

[25] Ministerio de Sanidad, Servicios Sociales e Igualdad (2015). Real Decreto 69/2015, de 6 de febrero, por el que se regula el Registro de Actividad de Atención Sanitaria Especializada (Spanish National Hospital Discharge Database). BOE.

[26] Gómez-Mayordomo V., Jiménez-García R., Zamorano-León J.J., Carabantes-Alarcón D., Bodas-Pinedo A., Hernández-Barrera V., López-de-Andrés A., Cuadrado-Corrales N. (2025). Demographic and Clinical Characteristics of Hospitalized Patients with Type 2 Diabetes Mellitus and Comorbid Parkinson’s Disease in Spain: A Nationwide Observational Study (2017–2023). J. Clin. Med..

[27] Quan H., Sundararajan V., Halfon P., Fong A., Burnand B., Luthi J.C., Saunders L.D., Beck C.A., Feasby T.E., Ghali W.A. (2005). Coding algorithms for defining comorbidities in ICD-9-CM and ICD-10 administrative data. Med. Care.

[28] Agencia Estatal Boletín Oficial del Estado (2007). Ley 14/2007, de 3 de julio, de Investigación biomédica. BOE.

[29] Ministerio de Sanidad, Consumo y Bienestar Social Solicitud de Extracción de Datos—Compromiso de Confidencialidad (Spanish National Hospital Discharge Database). https://www.mscbs.gob.es/estadEstudios/estadisticas/estadisticas/estMinisterio/SolicitudCMBDdocs/2018_Formulario_Peticion_Datos_RAE_CMBD.pdf.

[30] Sarica C., Conner C.R., Yamamoto K., Yang A., Germann J., Lannon M.M., Samuel N., Colditz M., Santyr B., Chow C.T. (2023). Trends and disparities in deep brain stimulation utilization in the United States: A Nationwide Inpatient Sample analysis from 1993 to 2017. Lancet Reg Health Am..

[31] Youngerman B.E., Chan A.K., Mikell C.B., McKhann G.M., Sheth S.A. (2016). A decade of emerging indications: Deep brain stimulation in the United States. J. Neurosurg..

[32] Buhmann C., Huckhagel T., Engel K., Gulberti A., Hidding U., Poetter-Nerger M., Goerendt I., Ludewig P., Braass H., Choe C.U. (2017). Adverse events in deep brain stimulation: A retrospective long-term analysis of neurological, psychiatric and other occurrences. PLoS ONE.

[33] Falowski S., Dierkes J. (2018). An Analysis of the Use of Multichannel Microelectrode Recording During Deep Brain Stimulation Surgeries at a Single Center. Oper. Neurosurg..

[34] Lee J.I. (2015). The Current Status of Deep Brain Stimulation for the Treatment of Parkinson Disease in the Republic of Korea. J. Mov. Disord..

[35] Hu W.H., Zhang K., Meng F.G., Ma Y., Zhang J.G. (2012). Deep brain stimulation in China: Present and future. Neuromodulation.

[36] Lopez Rios A.L., Piedimonte F., Arango G.J., Teixeira M.J., Arellano-Reynoso A., Del Carmen César G., Carmona H., Ciampi de Andrade D., Aníbal Restrepo-Bravo C., Gloria Escobar J.M. (2024). Deep brain stimulation in Latin America in comparison with the US and Europe in a real-world population: Indications, demographics, techniques, technology, and adverse events. J. Neurosurg..

[37] Santos-García D., Catalán M.J., Puente V., Valldeoriola F., Regidor I., Mir P., Matías-Arbelo J., Parra J.C., Grandas F. (2021). Continuous intestinal infusion of levodopa-carbidopa in patients with advanced Parkinson’s disease in Spain: Subanalysis by autonomous community. Neurol. (Engl. Ed.).

[38] DeLong M.R., Huang K.T., Gallis J., Lokhnygina Y., Parente B., Hickey P., Turner D.A., Lad S.P. (2014). Effect of advancing age on outcomes of deep brain stimulation for Parkinson disease. JAMA Neurol..

[39] Mathkour M., Garces J., Scullen T., Hanna J., Valle-Giler E., Kahn L., Arrington T., Houghton D., Lea G., Biro E. (2017). Short- and Long-Term Outcomes of Deep Brain Stimulation in Patients 70 Years and Older with Parkinson Disease. World Neurosurg..

[40] Olson M.C., Shill H., Ponce F., Aslam S. (2023). Deep brain stimulation in PD: Risk of complications, morbidity, and hospitalizations: A systematic review. Front. Aging Neurosci..

[41] Hariz G.M., Limousin P., Zrinzo L., Tripoliti E., Aviles-Olmos I., Jahanshahi M., Hamberg K., Foltynie T. (2013). Gender differences in quality of life following subthalamic stimulation for Parkinson’s disease. Acta Neurol. Scand..

[42] Hariz G.M., Nakajima T., Limousin P., Foltynie T., Zrinzo L., Jahanshahi M., Hamberg K. (2011). Gender distribution of patients with Parkinson’s disease treated with subthalamic deep brain stimulation; a review of the 2000–2009 literature. Parkinsonism Relat Disord..

[43] Martinez-Martin P., Falup Pecurariu C., Odin P., van Hilten J.J., Antonini A., Rojo-Abuin J.M., Borges V., Trenkwalder C., Aarsland D., Brooks D.J. (2012). Gender-related differences in the burden of non-motor symptoms in Parkinson’s disease. J. Neurol..

[44] Santos-García D., Laguna A., Hernández-Vara J., de Deus Fonticoba T., Cores Bartolomé C., Feal Painceiras M.J., Íñiguez-Alvarado M.C., García Díaz I., Jesús S., Boungiorno M.T. (2023). Sex Differences in Motor and Non-Motor Symptoms among Spanish Patients with Parkinson’s Disease. J. Clin. Med..

[45] Yoon J.E., Kim J.S., Jang W., Park J., Oh E., Youn J., Park S., Cho J.W. (2017). Gender Differences of Nonmotor Symptoms Affecting Quality of Life in Parkinson Disease. Neurodegener. Dis..

[46] Doshi P.K., Rai N., Das D. (2022). Surgical and Hardware Complications of Deep Brain Stimulation-A Single Surgeon Experience of 519 Cases over 20 Years. Neuromodulation.

[47] Fenoy A.J., Simpson R.K. (2014). Risks of common complications in deep brain stimulation surgery: Management and avoidance. J. Neurosurg..

[48] Boviatsis E.J., Stavrinou L.C., Themistocleous M., Kouyialis A.T., Sakas D.E. (2010). Surgical and hardware complications of deep brain stimulation. A seven-year experience and review of the literature. Acta Neurochir..

[49] Bjerknes S., Skogseid I.M., Sæhle T., Dietrichs E., Toft M. (2014). Surgical site infections after deep brain stimulation surgery: Frequency, characteristics and management in a 10-year period. PLoS ONE.

[50] Rolston J.D., Englot D.J., Starr P.A., Larson P.S. (2016). An unexpectedly high rate of revisions and removals in deep brain stimulation surgery: Analysis of multiple databases. Park. Relat. Disord..

[51] Abode-Iyamah K.O., Chiang H.Y., Woodroffe R.W., Park B., Jareczek F.J., Nagahama Y., Winslow N., Herwaldt L.A., Greenlee J.D.W. (2018). Deep brain stimulation hardware-related infections: 10-year experience at a single institution. J. Neurosurg..

[52] Bernstein J.E., Kashyap S., Ray K., Ananda A. (2019). Infections in Deep Brain Stimulator Surgery. Cureus.

[53] Chen T., Mirzadeh Z., Lambert M., Gonzalez O., Moran A., Shetter A.G., Ponce F.A. (2017). Cost of Deep Brain Stimulation Infection Resulting in Explantation. Stereotact. Funct. Neurosurg..

[54] Wetzelaer P., Vlis T., Tonge M., Ackermans L., Kubben P., Evers S., Kocabicak E., Temel Y. (2018). Management of Hardware Related Infections after DBS Surgery: A Cost Analysis. Turk. Neurosurg..

[55] Kähkölä J., Kähkölä J., Puhto T., Katisko J., Lahtinen M. (2024). Recommendations for the Prevention and Management of Deep Brain Stimulation Infections Based on 26-Year Single-Center Experience. Stereotact. Funct. Neurosurg..

[56] García-de Cruz S., Aldea-Mansilla C., Campos Bueno Á., Del Villar Sordo V. (2018). Reliability of the Minimum Basic Data Set as an Epidemiological Tool in Tuberculosis. Arch Bronconeumol. (Engl. Ed.).

[57] Xu S.S., Malpas C.B., Bulluss K.J., McDermott H.J., Kalincik T., Thevathasan W. (2022). Lesser-Known Aspects of Deep Brain Stimulation for Parkinson’s Disease: Programming Sessions, Hardware Surgeries, Residential Care Admissions, and Deaths. Neuromodulation.

[58] Verla T., Marky A., Farber H., Petraglia F.W., Gallis J., Lokhnygina Y., Parente B., Hickey P., Turner D.A., Lad S.P. (2015). Impact of advancing age on post-operative complications of deep brain stimulation surgery for essential tremor. J. Clin. Neurosci..

