# Supplementary material for: Deep Brain Stimulation for Movement Disorders in Spain: Temporal Trends, Complications, and Sex-Related Disparities (2002–2019)
Source: Healthcare (Basel). 2026 Mar 6;14(5):672. doi: 10.3390/healthcare14050672 (PMC12984668; doi:10.3390/healthcare14050672)
Supplement: Supplementary file 1 [file healthcare-14-00672-s001.zip › healthcare-4106663-supplementary.pdf]

Table S1. ICD9 and ICD10 codes for diagnosis, procedures and complications used in this investigation.

	ICD9	ICD10
Parkinson's disease	332.0	G20
Essential tremor	333.1	G25.0
Dystonia	333.6, 333.7, 333.8	G24
DBS electrode implantation	02.93	00H00MZ, 00H03MZ 00H04MZ
DBS electrode explantation / removal/ surgical revision	01.22	00P00MZ, 00P03MZ, 00P04MZ 00P0XMZ
Mechanical failure of brain neurostimulator electrode (Wire)	996.2	T85.110A, T85.110D, T85.110S, T85.113A, T85.113D, T85.113S
Electrode Malposition (Wire)	996.2	T85.120A, T85.120D, T85.120S, T85.123A, T85.123D, T85.123S
Electrode rupture	996.2	T85.190A, T85.190D, T85.190S
Device infection	996.63	T85.731A, T85.734A, T85.738A
Intracranial hemorrhage	430, 431, 432	I60, I61, I62
CNS infection	320, 321, 322, 323, 324, 325, 326	G00, G01, G02, G03, G04, G05, G06, G07, G08, G09
Postoperative wound infection	998.5	T81.4
Sepsis:	995.9	R65
Ischemic stroke:	433, 434, 436. 362.3	I63, I65, I66, I67, I68, I69
Pneumonia	480–488, 507	J09-J18, J69.0
Deep vein thrombosis	453.4, 453.5, 453.6, 453.7, 453.8	I82.4, I82.5, I82.6, I82.7, I82. A, I82. B, I82. C, I82.8, I82.9
Pulmonary thromboembolism	415.1	I26
Acute renal failure	584	N17
Delirium	293	F05
Epileptic seizures	345	G40
Blood transfusion	99.01, 99.02, 99.03, 99.04, 99.05, 99.06, 99.07, 99.08, 99.09	30233H0 30233N0 30243H0 30243N0 30233H1 30243H1 30233N1 30233P1 30243N1 30243P1 30233R1 30243R1 30233T1 30233V 30233W1 302433W1 302433P1 T1 30243V1 30243W1 30233J1 30233K1 30233L1 30233M1 30243J1 30243K1 30243L1 30243M1 3E033GC 3E043GC 3E053GC 3E063GC 30233Q1 30243Q1
Mechanical ventilation	96.7	5A1945Z, 5A1955Z, 5A1935Z, 5A09357, 5A09457, 5A09557

Table S2. Postoperative complications for deep brain stimulation electrode implantations admissions in movement disorders in Spain according to diagnosis (2002-2019).

Total. N (%)	Parkinson´s disease (2002-2019)	Essential tremor (2002-2019)	Dystonia (2002-2019)	P Essential tremor vs. Parkinson´s disease	P Essential tremor vs. Dystonia	P Parkinson´s disease vs Dystonia
Electrode malposition/ mechanical failure	226 (5.61)	28 (5.14)	11 (3.58)	0.654	0.133	0.297
Device infection	64 (1.59)	11 (2.02)	2 (0.65)	0.457	0.196	0.118
Intracranial haemorrhage	31 (0.77)	7 (1.28)	2 (0.65)	0.213	0.819	0.386
CNS infection	5 (0.12)	0 (0)	1 (0.33)	0.411	0.359	0.182
Postoperative wound infection	23 (0.57)	2 (0.37)	2 (0.65)	0.545	0.857	0.56
Sepsis	2 (0.05)	1 (0.18)	0 (0)	0.252	0.696	0.453
Pneumonia	12 (0.3)	5 (0.92)	3 (0.98)	0.026*	0.051	0.931
Deep vein thrombosis	4 (0.1)	0 (0)	0 (0)	0.462	0.581	
Pulmonary thromboembolism	4 (0.1)	0 (0)	0 (0)	0.462	0.581	
Acute renal failure	6 (0.15)	0 (0)	0 (0)	0.367	0.499	
Delirium	33 (0.82)	2 (0.37)	0 (0)	0.256	0.112	0.288
Epileptic seizures	36 (0.89)	7 (1.28)	9 (2.93)	0.374	0.001*	0.089
Blood transfusion	17 (0.42)	4 (0.73)	9 (2.93)	0.311	0	0.012*
Mechanical ventilation	35 (0.87)	3 (0.55)	8 (2.61)	0.443	0.003*	0.011*

CNS Central nervous system

Table S3. Postoperative complications for deep brain stimulation explantations/revisions admissions in movement disorders in Spain (2002-2019).

	**Parkinson´s disease (2002-2019)**	**Essential tremor (2002-2019)**	**Dystonia (2002-2019)**	**P Essential tremor vs. Parkinson´s disease**	**P Essential tremor vs. Dystonia**	**P Parkinson´s disease vs Dystonia**
Electrode malposition/ mechanical failure. N (%)	113 (26.59)	8 (21.05)	4 (11.76)	0.457	0.056	0.291
Device infection. N (%)	144 (33.88)	10 (26.32)	11 (32.35)	0.343	0.856	0.574
Intracranial haemorrhage. N (%)	2 (0.47)	0 (0)	0 (0)	0.672	0.689	
CNS infection. N (%)	8 (1.88)	1 (2.63)	0 (0)	0.749	0.42	0.341
Postoperative wound infection. N (%)	33 (7.76)	2 (5.26)	4 (11.76)	0.576	0.41	0.319
Sepsis. N (%)	0 (0)	0 (0)	0 (0)			NA
Pneumonia. N (%)	4 (0.94)	0 (0)	0 (0)	0.548	0.57	
Deep vein thrombosis. N (%)	1 (0.24)	1 (2.63)	0 (0)	0.031	0.777	0.341
Pulmonary thromboembolism. N (%)	1 (0.24)	1 (2.63)	0 (0)	0.031	0.777	0.341
Acute renal failure	2 (0.47)	0 (0)	0 (0)	0.672	0.689	NA
Delirium	1 (0.24)	0 (0)	0 (0)	0.765	0.777	NA
Epileptic seizures	4 (0.94)	0 (0)	0 (0)	0.548	0.57	NA
Blood transfusion	0 (0)	1 (2.63)	1 (2.94)	0.001	0	0.936
Mechanical ventilation	2 (0.47)	0 (0)	0 (0)	0.672	0.689	NA

CNS Central nervous system
